# Facile Synthesis of g-C_3_N_4_/MoO_3_ Nanohybrid for Efficient Removal of Aqueous Diclofenac Sodium

**DOI:** 10.3390/nano11061564

**Published:** 2021-06-14

**Authors:** Jamshaid Rashid, Faryal Saleemi, Bilal Akram, Lin Wang, Naveed Hussain, Ming Xu

**Affiliations:** 1 College of Environment and Planning, Henan University, Kaifeng 475004, China; jamshaidrashid@gmail.com; 2 Key Laboratory of Geospatial Technology for the Middle and Lower Yellow River Regions, Ministry of Education, Henan University, Kaifeng 475004, China; 3 Department of Environmental Science, Faculty of Biological Sciences, Quaid-I-Azam University, Islamabad 45320, Pakistan; faryalsaleemi@gmail.com; 4 Department of Chemistry, Tsinghua University, Beijing 100084, China; bai-l16@tsinghua.org.cn; 5 Miami College, Henan University, Kaifeng 475004, China; 6 Institute of Fundamental and Frontier Sciences (IFFS), University of Electronic Science and Technology of China, Chengdu 610054, China; n.hussain7766@gmail.com; 7 Henan Key Laboratory of Earth System Observation and Modeling, Henan University, Kaifeng 475004, China

**Keywords:** diclofenac sodium, efficient adsorbent, g-C_3_N_4_, micropollutants, nanohybrid

## Abstract

Graphitic carbon nitride modified by molybdenum trioxide (g-C_3_N_4/_MoO_3_) as a nanohybrid was synthesized by co-precipitation method. Here, g-C_3_N_4_/MoO_3_ nanohybrid was used for the first time as an adsorbent for the pharmaceutical drug, diclofenac, (an aqueous micropollutant) from water to mitigate its possible environmental toxic effects. Compared to pristine components, the nanohybrid exhibited better adsorptive removal of diclofenac. Adsorption was enhanced with increment in MoO_3_ content from 1 to 3 wt %; however further increment in MoO_3_ content resulted in lower adsorption capacity due to agglomeration of MoO_3_ particles over g-C_3_N_4_. 162 mg g^−1^ adsorption capacity was achieved for 300 mg L^−1^ diclofenac in solution with 1 g L^−1^ adsorbent at pH = 6. Adsorption of diclofenac over g-C_3_N_4_ /MoO_3_ followed pseudo 2nd order kinetics. Temkin, Langmuir, Dubinin Radushkevich and Freundlich isotherm models were applied on the experimental results concluding that diclofenac adsorption over g-C_3_N_4_/MoO_3_ followed the Langmuir isotherm. The adsorption mechanism could be explained by the π–π interaction between aromatic rings of diclofenac and g-C_3_N_4_/MoO_3_ (3%) nanohybrid, which is also evident by the FTIR results. This study presents the facile fabrication of a 2nd generation adsorbent for the treatment of diclofenac contaminated water that may as well help achieve the removal of other micropollutants form water.

## 1. Introduction

Pharmaceuticals are extensively used all over the world to cure, impede, and treat diseases in both humans and animals. Globally, an annual per capita of 15 g of pharmaceutical drugs are consumed, while in industrialized countries this consumption rate is estimated at between 50 and 150 g [1]. Among pharmaceuticals, diclofenac sodium—2-(2-(2,6-dichloroanilino) phenyl) acetic acid—is most frequently administered for pain relief from rheumatoid arthritis, post-operative pain and the chronic pain regularly associated with cancer [1]. McGettigan and Henry stated that diclofenac is prominently known as the world’s most popular painkiller by means of its extensive usage in human medical care with yearly consumption of between 195 and 940 mg per individual [2]. Estimation of health data from 86 countries showed that approximately 1309 tonnes of diclofenac is being consumed globally; where Asia accounts for 40% of consumption and Europe 29%, excluding the veterinary consumption [3]. Such huge consumption of diclofenac has resulted in its frequent release into the environment from distinct routes such as through untreated wastewater, direct discarding of unused or expired medication, excretion of the human and veterinary drugs as metabolites or unchanged compounds and discharge from pharmaceutical manufacturing plants [4]. In various countries diclofenac and its metabolites were observed from a few ng to μg L^−1^. Concentrations such as 380 ng L^−1^ are documented for German and Spanish groundwaters [5]. Up to 95 and 148 μg L^−1^ diclofenac was detected in the bile of two freshwater fish species, which was approximately 1000 times larger than aqueous concentrations [6]. Diclofenac was also identified within the tissues of wild mussels sampled from non-contaminated tourists spot. Among terrestrial animals, almost 98% of the population decline of three Gyps vulture species in Pakistan, India, and Bangladesh was due to diclofenac poisoning [7,8]. Most of the studies direct to the possibility of renal failure because of scavenging on livestock having residues of diclofenac [9]. Oaks and co-researchers, very firstly, studied the relationship between diclofenac residue and vulture population decline. Autopsy showed the presence of 0.120–0.906 µg g^−1^ of diclofenac residues in the kidneys of vultures [10]. Subsequently, IUCN included these vulture species in their list of “critically endangered” species [11]. Owing to the toxic effects of diclofenac and occurrence in various waterbodies, “European Water Framework Directive” has classified diclofenac as a priority pollutant. A revision of this document in 2013 resulted in inclusion of diclofenac in the watch list of substances monitored on priority basis for the determination of risk reduction measures. The Environmental Quality Standards (EQS) for Europe has defined average permissible concentration of 0.1 μg L^−1^ in freshwater [12]. To maintain allowable concentrations of diclofenac, desirable treatment technologies are needed. In this context, a variety of techniques have been utilized including microbial degradation, advanced oxidation processes, membrane bioreactors (MBR) and granular activated carbon (GAC) [13,14,15,16,17]. Their efficient usage is hindered by either formation of transformation by products [18] excessive time span requirement or prerequisite of high energy and subsequently higher cost [19]. Currently, among different suggested techniques, adsorption provides high efficacy, simplicity and incorporation into existing wastewater remediation processes. Recently, numerous kinds of adsorbents were utilized for diclofenac adsorption such as zeolites, organoclays, variety of activated carbons, metal organic frameworks and molecularly imprinted polymers [17,20]. Despite the manifestation of worthy performances, these adsorbents either suffer from uncommendable efficacy to remove micropollutants or regeneration and economic feasibility halt their employment at a larger scale.

Recently, the application of nano-scale materials as an adsorbent has attracted attention for removing toxic and hazardous contaminants from wastewater [21]. Among numerous nanomaterials, the graphitic carbon nitride (g-C_3_N_4_) exhibiting excellent chemical and thermic stability in ambient environments and without any ecotoxicity [22]. The performance of g-C_3_N_4_ as an adsorbent can be markedly improved through modification of surface properties by adding chemical groups, surfactants, polymers, etc. Kumar and co-workers oxidized the g-C_3_N_4_ to enhance its specific surface area and further functionalized it with polyaniline nanofiber to provide positive surface charge and to bind anionic chromium ions electrostatically [23]. Zou’s group synthesized β-cyclodextrin modified g-C_3_N_4_ for adsorptive separation of lead from aqueous solutions. In their study, surface complexation was suggested for the adsorption of lead whereas π–π interaction and hydrogen bonding was responsible for the adsorption of methyl orange onto the functionalized adsorbent [24]. Transition metals have been utilized in the wastewater treatment methods by incorporating them into other organic or inorganic materials. Selective but fast adsorption of organic contaminants has been displayed by ordered microstructures such as molybdenum trioxide (MoO_3_). MoO_3_ has been found very useful in numerous research and commercial applications due to its unique catalytic and electrochemical characteristics. However, a limited number of research studies have explored their use as an adsorbent [25,26]. For instance, up to 97.9% removal of 20 mg L^−1^ rhodamine B was achieved by MoO_3_ within 10 min, which is expressively fast with a maximum removal capacity 9-times higher than commercial activated carbon [27]. Nanocrystalline α-MoO_3_, as a nanosorbent for methylene blue exhibited a removal capacity of 152 mg/g [28]. Similarly, 98% of 20 mg L^−1^ MB was adsorbed from water in nearly 25 min without the aid of light [29]. Furthermore, the well-organized heptazine units of g-C_3_N_4_ contains numerous “nitrogen pots” as they hold 6 N e^−^ providing functionalities with negative charges. The electrons in the aromatic ring offer metal inclusion in these nitrogen pots [30]. Molybdenum doped g-C_3_N_4_ photocatalyst has been synthesized by Wang and coworkers. The BET results indicated that with the increase in molybdenum concentrations, from 1–5% enhanced the catalyst’s surface area up to 88 m^2^ g^−1^. Increase in molybdenum content impeded the crystal growth of g-C_3_N_4_, leading the crystalline structure of g-C_3_N_4_ towards amorphousness, hence increasing the porosity of the material [26]. In another study, calcined form of commercial silica–alumina and impregnated oxides of molybdenum, cerium, and nickel were assessed as adsorbents for the removal of sulfur and nitrogen compounds from a hydro-treated Brazilian diesel. Among all metal oxide functionalized adsorbents, incorporation of molybdenum oxide led to an increase of the adsorptive capacities of sulfur and nitrogen per specific area [31]. Therefore, in this study, g-C_3_N_4_ modified with MoO_3_ was utilized for enhanced adsorption of diclofenac micropollutant.

## 2. Materials and Methods

### 2.1. Chemicals

Ammonium heptamolybdate ((NH_4_)_6_Mo_7_O_24_), hydrochloric acid (HCl 37%), sodium hydroxide (NaOH), ethanol (C_2_H_6_O) and diclofenac sodium (C_14_H_10_Cl_2_NNaO_2_) were purchased from Sigma Aldrich (St. Louis, MO, USA), Urea (CO(NH_2_)_2_) was bought from Merck (Darmstadt, Germany). All chemicals used were of high purity (i.e., 98–99%).

### 2.2. Synthesis of Nanohybrid (g-C_3_N_4 (1-x)_/MoO_3(x)_)

To prepare nanohybrids, the g-C_3_N_4_ was modified with molybdenum trioxide. Molybdenum trioxide was separately prepared by solid state decomposition method. Ten grams of (NH_4_)_6_Mo_7_O_24_ was decomposed at 500 °C (for 4 h). The final grey colored powder was washed and then dried (at 60 °C). Nanohybrids of g-C_3_N_4_/MoO_3_ were synthesized by modification in the procedure already described [32]. Firstly, different weight percentages of MoO_3_ (1%, 3%, 5%, 7%) were added to the g-C_3_N_4_ in deionized water and then under constant stirring the deionized water was evaporated from the mixture by using hotplate. The slurry thus obtained was dried at 80 °C and ground using a pestle and mortar. Calcination of this ground powder at 300 °C for 3 h in a muffle furnace gave the final g-C_3_N_4_/MoO_3_ nanohybrids.

### 2.3. Characterization

The crystallinity of the synthesized nanomaterials was analyzed by Burker D8 Diffractometer (Madison, WI, USA) using Cu Kα radiations (λ = 1.5418 Å) and incident angle (2θ) ranged between 10–80°. XPS measurements were performed using standard Omicron system (Uppsala, Sweden) equipped with monochromatic Al Kα 1486.7 eV (at 15 keV and energy 100 eV or 20 eV). Morphological studies were conducted by JEOL-2100 (Peabody, MA, USA) Transmission Electron Microscope (TEM). FTIR spectroscopic analysis demonstrated existence of functional groups of as synthesized and used adsorbents. For this purpose, the IR spectrum from 4000 to 400 cm^−1^ was noted by a FTIR spectrophotometer (Bruker, Tensor 27 (Madison, WI, USA)).

### 2.4. Adsorption Assays

Adsorption experiments for the synthesized nanocomposites were performed for the aqueous pharmaceutical drug, diclofenac sodium, as a model micropollutant. For typical experiments 0.1 g of adsorbent was added to the 100 mL working solution of diclofenac sodium continuously stirred for 3 h at 300 rpm. After regular time gaps 5 mL aliquots were strained through a 0.45 µm syringe filter before storing the samples into amber glass vials. The residual concentrations of diclofenac were quantitatively analyzed by using a UV 3000 spectrophotometer (Los Angeles, CA, USA) at a λ_max_ of 276 nm. 

## 3. Results and Discussion

### 3.1. Material Characterizations

**Figure 1:**
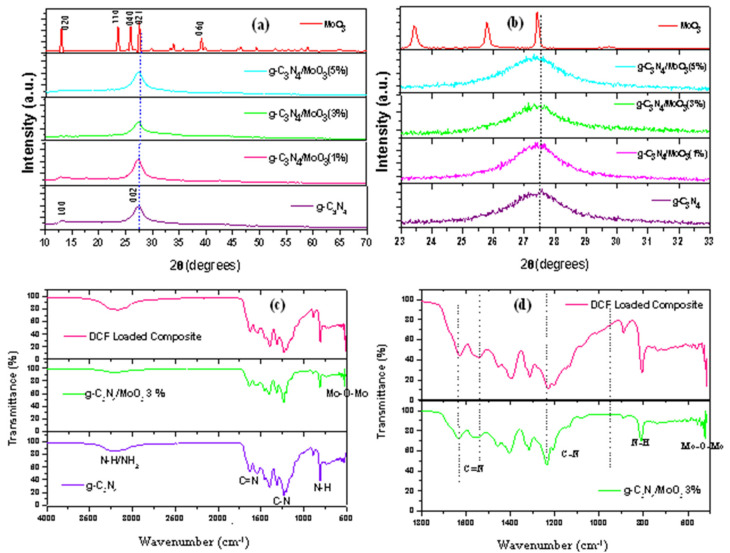
(**a**) XRD spectra and (**b**) resolved XRD of components and binary g-C_3_N_4_/MoO_3_ nanohybrids; (**c**) FTIR Spectra and (**d**) resolved FTIR of as prepared and diclofenac loaded nanohybrid.

The XRD peaks of pristine components and binary nanocomposites are illustrated in Figure 1a. The strong characteristic peak of pure g-C_3_N_4_ appears at 27.5° which correspond to (002) plane of g-C_3_N_4_, pointing out the existence of inter-planar layering of conjugated aromatic carbon nitride units with interlayer spacing of 0.33 nm. The small intensity peak 13.2° is attributed to (100) in-plane repeated heptazine in g-C_3_N_4_ structure having in-plane d spacing = 0.67 nm. The orthorhombic phase of MoO_3_ can be indexed from the XRD patterns which are in accordance with the JCPDS card no 05-0508. The main peaks observed at 12.7°, 23.33°, 25.7°, 27.38° and 38.98° represent (020), (110), (040), (021) and (060) planes, respectively [31]. As shown in Figure 1b, increase in MoO_3_ content in the g-C_3_N_4_/MoO_3_ composites from 1 to 5% displayed slight shift in g-C_3_N_4′_s characteristic peak at 27.5° towards MoO_3′_s highest intensity peak at 27.38° indicating deposition of MoO_3_ did not affect the tri-s-triazine structure of g-C_3_N_4_ sheets.

The FTIR spectrum in Figure 1c represents a characteristic band of g-C_3_N_4_ at 807 cm^−1^, attributed to the vibration of tri-s-triazine ring. Similarly, the 890 cm^−1^ band could be assigned to N–H deformation mode while 1100–1700 cm^−1^ band corresponds to stretching mode of bridging C–NH–C units. Vibrations at 1133 cm^−1^ could be ascribed to C–N stretching of an aromatic amine whereas peaks at 1630 and 1538 cm^−1^ may correspond to aromatic C=N stretches and peaks at 1312 and 1230 cm^−1^ attributed to C–N stretching vibrations. The broad bands in the range of the 3000–3300 cm^−1^ region is assigned to the adsorbed N–H and/or NH_2_ vibration. Peak intensity at 556–569 cm^−1^ originated from bending vibration of the Mo–O–Mo unit where oxygen ions are linked to Mo^6+^; describing deposition of MoO_3_ onto g-C_3_N_4_ [27]. Broadening in the 1133–1312 cm^−1^ stretching vibrations and larger band adsorption at the 3000–3300 cm^−1^ region are observed in Figure 1d. Diclofenac loaded g-C_3_N_4_/MoO_3_ (3%) sample indicates π–π stacking of diclofenac onto the g-C_3_N_4_/MoO_3_ nanohybrid. Moreover, the available hydrogen bonding sites (–NH) also tend to increase adsorption of the polar diclofenac molecule [33], suggesting that π–π interaction and hydrogen bonding is responsible for the adsorption of diclofenac onto the g-C_3_N_4_/MoO_3_ (3%) nanohybrid. 

**Figure 2:**
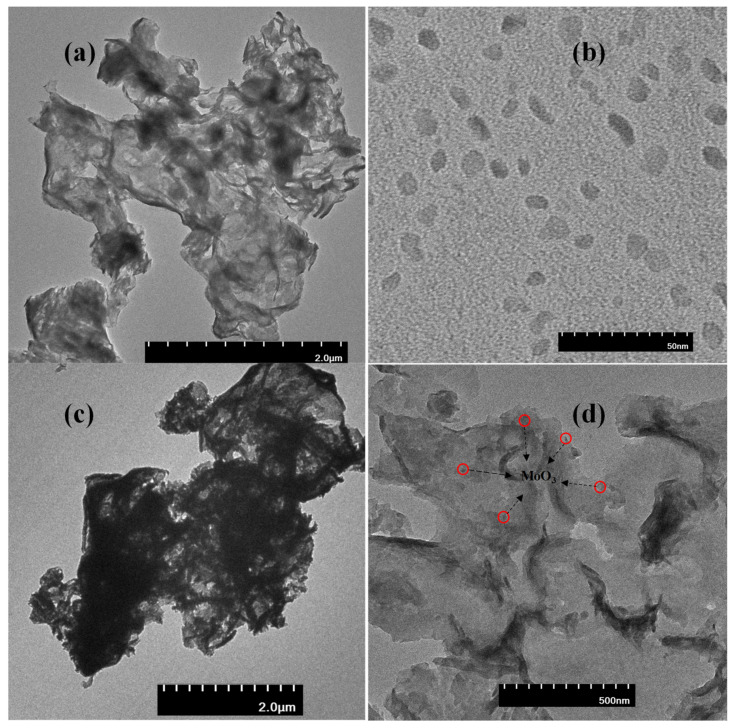
Microscopic image of (**a**) g-C_3_N_4_, (**b**) MoO_3_ and (**c**,**d**) g-C_3_N_4_/MoO_3_ (3%) nanohybrid.

For clarification of nanohybrid morphology and particle dimensions, the synthesized materials were subjected to TEM imaging (Figure 2). The g-C_3_N_4_ appeared to comprise of a few layered nanosheets indicating the aggregation of synthesized nanosheets (Figure 2a), while the MoO_3_ nanoparticles appear to be of nearly 25 nm dimensions (Figure 2b). The TEM images of the nanohybrid (Figure 2c,d) show nanosized MoO_3_ particles dispersed randomly over the darkened nanosheets of g-C_3_N_4_.

**Figure 3:**
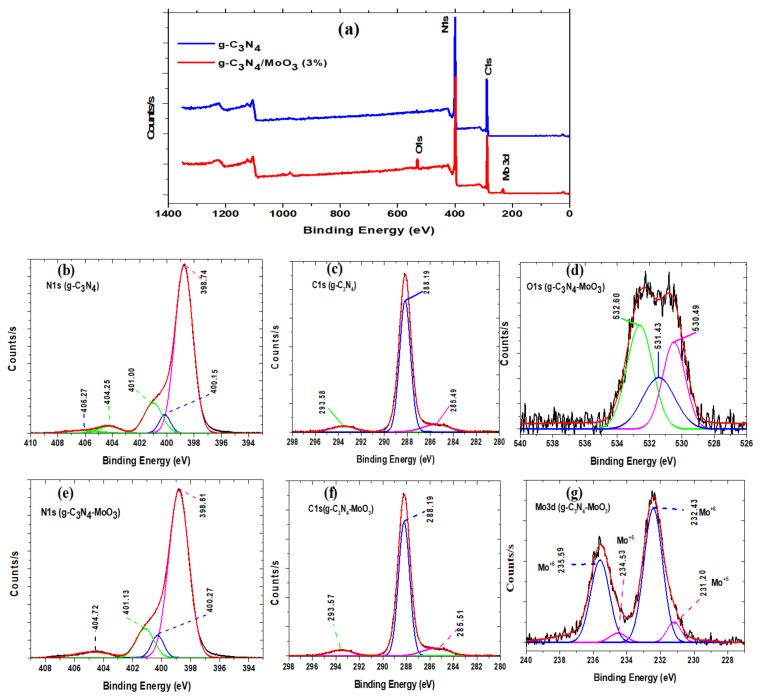
(**a**) XPS scan of gC_3_N_4_ and g-C_3_N_4_/MoO_3_(3%) and (**b**–**g**) resolved XPS for N1s, C1s, O1s and Mo3d.

The chemical structures of pristine g-C_3_N_4_ and g-C_3_N_4_/MoO_3_ (3%) nanohybrid analyzed by XPS is provided in Figure 3. The survey scan of g-C_3_N_4_ and g-C_3_N_4_/MoO_3_ (3%) comprising of C, N and C, N, O, Mo, respectively. The N1s spectra of g-C_3_N_4_ was resolved into three typical peaks at 398.7, 400.2 and 401.0 eV (Figure 3b), representing N-*sp^2^*C, C-N–H, and N-(C)_3_ bonds, respectively [34,35]. In case of the g-C_3_N_4_/MoO_3_ nanohybrid (Figure 3e), the characteristic peaks shifted slightly to higher energy at 398.8, 400.3 and 401.1 eV indicating interaction between MoO_3_ and g-C_3_N_4_. Resolved spectra of g-C_3_N_4_ shows C1s peaks at 285.5 eV and 288.2 eV (Figure 3c), indicating surface bound carbon and N=C-N bond, respectively [36]. A slight shift in the C1s peak in case of g-C_3_N_4_/MoO_3_ nanohybrid from 285.5 to 285.5 eV suggests incorporation of MoO_3_ over g-C_3_N_4_ nanosheets (Figure 3f). 

The resolved spectra of O1s for g-C_3_N_4_/MoO_3_ (3%) is shown in Figure 3d, comprising of three peaks observed at 530.5, 531.4 and 532.6 eV corresponding to Mo–O, oxygen vacancies and surface hydroxyl groups, respectively [37]; while, the resolved spectra of Mo 3d for g-C_3_N_4_/MoO_3_ (3%) showed two types of peaks located at 232.4 and 235.6 eV corresponding to Mo3d^5/2^ and Mo3d^3/2^ for Mo^6+^ in MoO_3_ Mo^6+^, while other peaks at 231.2 and 234.5 eV correspond to Mo^5+^ [16,38]. The mixed valence states of Mo cations are indicative of surface oxygen vacancies [39].

### 3.2. Adsorption Activities

#### 3.2.1. Effect of g-C_3_N_4_ Modification on Diclofenac Removal

For the adsorptive removal of diclofenac, pristine nanomaterials and their modified forms were scrutinized. All experiments were performed at normal solution pH 6 with Diclofenac 25 mg L^−1^. The best performing nanohybrid was selected by examining the removal efficiency of g-C_3_N_4_/MoO_3_ nanohybrids. The base material, g-C_3_N_4_ and MoO_3_, exhibited 23% and 65% removal within first 15 min of the adsorption process and no further removal was observed for the remaining 175 min. g-C_3_N_4_/MoO_3_ nanohybrids showed prominent adsorptive removal of 44%, 83%, 71% and 68% for 1%, 3%, 5% and 7% MoO_3_ loaded g-C_3_N_4_, respectively. The better adsorption of diclofenac on to different weight percentages of g-C_3_N_4_/MoO_3_ nanohybrids as compared to base materials suggested that MoO_3_ was completely deposited on g-C_3_N_4_ nanosheets. These results are supported by X-ray photoelectron spectroscopy and also by another study suggesting enhanced adsorption shown by the composites may be attributed to the presence of oxygen vacancies, hydroxyl groups, and molecular water on the MoO_3_ surface [40]. Studies suggest that the increase in molybdenum content may have impede the crystal growth of g-C_3_N_4_; leading crystalline structure of g-C_3_N_4_ towards amorphousness, hence, increasing porosity of the material [26]. Due to better performance of g-C_3_N_4_/MoO_3_ (3%) among all adsorbents, it was chosen for further optimization studies.

#### 3.2.2. Diclofenac Concentration and Solution pH Effect on Adsorption

**Figure 4:**
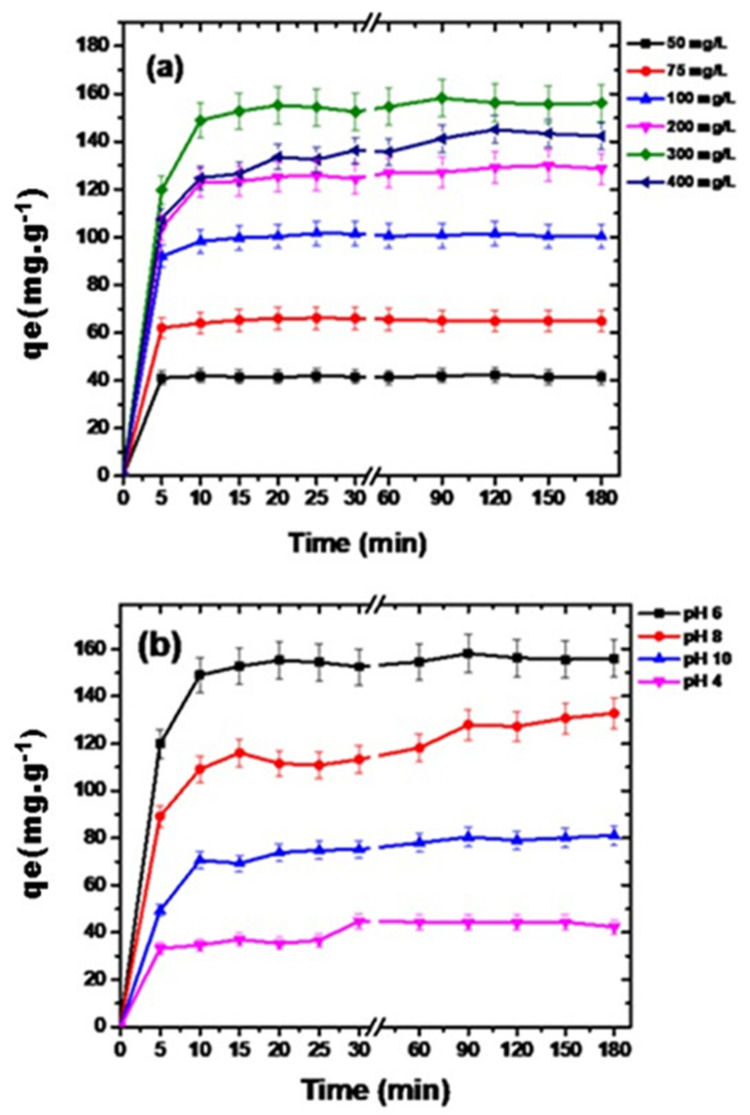
Variation in adsorption capacity of g-C_3_N_4_/MoO_3_ (3%) over (**a**) changing concentrations of aqueous diclofenac; (**b**) solution pH.

Pollutant concentration plays a vital role in determining adsorbent’s removal efficiency. Adsorption capacity of g-C_3_N_4_/MoO_3_ (3%) is influenced by the initial concentrations of diclofenac. As depicted in Figure 4a, increase from 50 to 100 mg L^−1^ diclofenac showed an increase in percent removal from 83% to 91%, but further increase in concentration resulted in a decrease in percent of removal efficiency. Initially, at lower starting concentrations, a large number of unoccupied reactive sites were accessible to diclofenac molecules for adsorption, so removal rate was higher. With increased diclofenac concentrations from 200 to 400 mg L^−1^, inadequate active sites were available for a large sum of molecules. This initiated a competition between excessive molecules of diclofenac over limited binding sites of adsorbent (i.e., catalyst dose = 1 g L^−1^); consequently, decline in adsorptive removal was seen. However, the adsorption capacity of g-C_3_N_4_/MoO_3_ (3%) enhanced with initial diclofenac concentration. An increment in adsorbate dose from 50 to 300 mg L^−1^ exhibited enhancement in adsorption capacity from 41.29 to 156.29 mg g^−1^. Here, rise in initial adsorbate concentration may have acted as a driving force for transfer of mass from surface to the inner pores, therefore increase in adsorption capacity was observed [39]. However, a further spike up to 400 mg L^−1^ indicated decrease in adsorption capacity indicating saturation of adsorbent.

Figure 4b, show how pH influences the diclofenac adsorption by g-C_3_N_4_/MoO_3_ (3%). Adsorbent and adsorbate surface chemistry and charge is greatly altered by change in pH of the solution by protonation and deprotonation. In water, diclofenac exists in two species—either neutral or in anionic form. In extreme acidic conditions it exists in neutral form but at pH above its pKa value (i.e., 4.1) anionic species is dominant. As in this study, at pH 4 initial diclofenac concentration in solution of 300 mg L^−1^ was reduced to 44 mg L^−1^ while the solution became turbid which indicated insolubility of diclofenac at acidic pH. The higher adsorption capacities of 158 and 132 mg g^−1^ at pH 6 and 8, respectively showed that diclofenac removal from aqueous solution was not dependent on electrostatic interaction. This appreciable adsorption of diclofenac could be explained by hydrogen bonding and π–π interaction between aromatic rings of diclofenac and g-C_3_N_4_/MoO_3_ (3%) composite [41].

#### 3.2.3. Effect of Adsorbent Dose and Temperature

**Figure 5:**
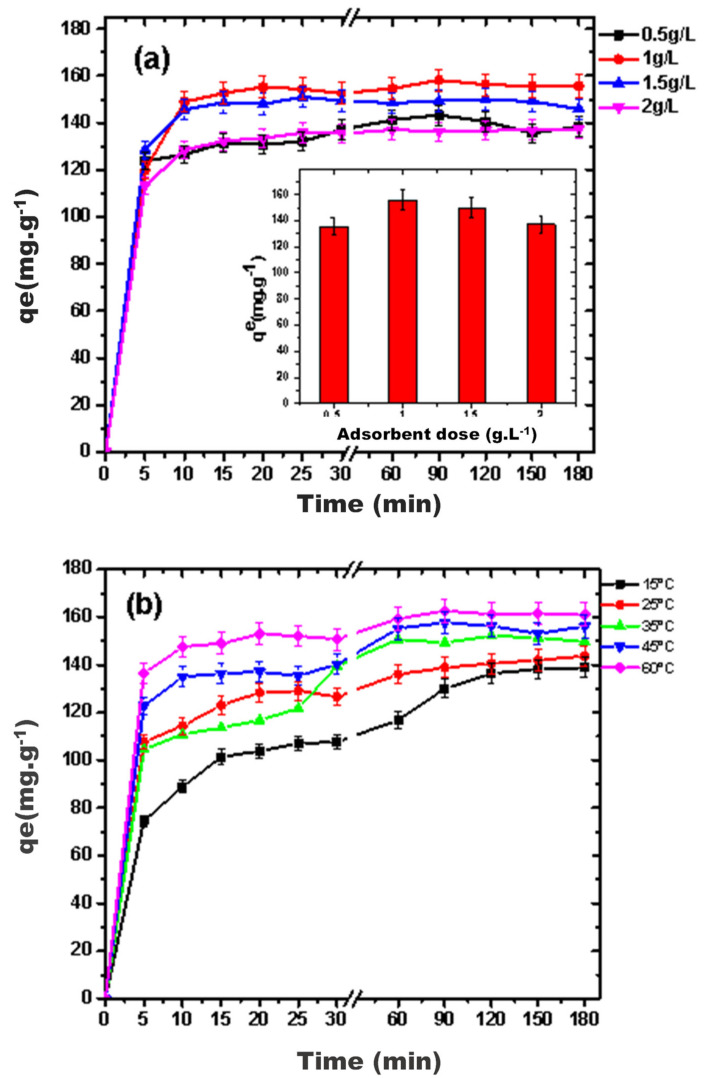
Variation in adsorption capacity of g-C_3_N_4_/MoO_3_ (3%) over (**a**) adsorbent concentration (**b**) thermal changes.

An adsorbent dose of 1 g L^−1^ was optimum for maximum adsorption (Figure 5a). Further increase in adsorbent concentration has a negative effect on adsorption due to adsorbent agglomeration. Variation in adsorption of diclofenac over g-C_3_N_4_/MoO_3_ (3%) nanohybrid was studied at 15, 25, 35, 45 and 60 °C using initial diclofenac concentration 300 mg L^−1^. The results illustrated in Figure 5b reveal that the solution temperature was directly proportional to the diclofenac % removal efficiency and adsorption capacity; indicating that adsorption process was endothermic. As the temperature was varied from 15 to 60 °C in series of experiments, the percent removal efficiency of diclofenac increased from 46.8% to 54% with every rise in temperature and in parallel adsorption capacity enhanced from 138.8 to 162.6 mg g^−1^. At higher temperatures, expansion within pores and activation of the adsorbent external surface brings about the availability of a greater sum of active sites, therefore, enhancing the adsorption of micropollutants. Moreover, at elevated temperatures the mobility of adsorbate increases while the viscosity of the solution decreases, thus, resulting in effective removal of the contaminant [42].

#### 3.2.4. Reaction Kinetics 

**Figure 6:**
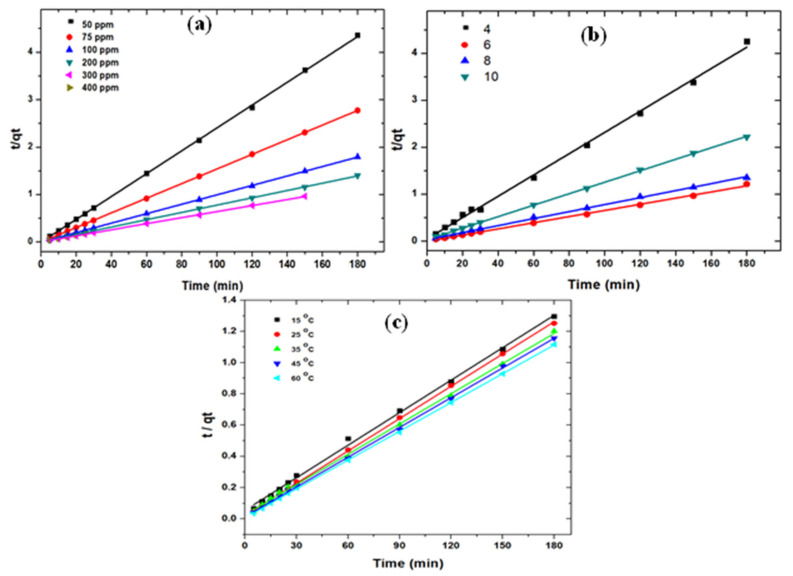
Pseudo 2nd order kinetic plots showing influence of varied (**a**) adsorbate concentration, (**b**) pH and (**c**) temperature on diclofenac adsorption over g-C_3_N_4_/MoO_3_ (3%) nanohybrid.

The rate of diclofenac uptake on g-C_3_N_4_/MoO_3_ (3%) nanohybrid could be explained by reaction kinetics. The pseudo 1st and 2nd order kinetics were used for better insight of the rate of diclofenac uptake by the 3%MoO_3_/g-C_3_N_4_ nanohybrid (Figure 6). The pseudo 1st order kinetic model assumes that adsorbate uptake is directly related to difference of saturation concentration and amount of adsorbate uptake with time; whereas, the pseudo 2nd order kinetic model suggests adsorption rates depend on the rate of adsorbate diffusion in the adsorbent pores [43]. The equations for both kinetic models are:

(1) $$\log(q_{e}-q_{t})={\;\mathrm{logq}}_{e}-(\frac{\mathrm{Kt}}{2.303})$$

(2) $$t/q_{t}=\left(1/{\mathrm{Kq}}_{e}^{2}\right)+\;t/q_{e}$$

K_1_ (min^−1^) and K_2_ (g mg^−1^ min^−1^) represent rate constants of pseudo 1st and pseudo 2nd order kinetic equations; q_e_ (mg g^−1^) equilibrium adsorption capacity and q_t_ (mg g^−1^) refers to temporal adsorption capacity.

The adsorption rate K_2_ depends on the operating conditions such as solution adsorbate concentrations, pH, and temperature as evident by Figure 6a–c, respectively. Results suggest that experimental data aligned well with pseudo 2nd order kinetics. The values of qe, K_1_, and K_2_ were calculated from the intercept and slope of the kinetic plots and listed in the Table 1. In the current study the value of adsorption rate coefficient, K_2,_ decreased from 0.0241 min^−1^ to 0.0062 min^−1^ with increasing diclofenac concentration from 50 to 300 mg L^−1^. Furthermore, the calculated adsorption capacities (qe_cal_) were in very close agreement to the experimental adsorption capacities. Similarly to the initial concentration, other operating parameters also exhibited trends indicating that the adsorption process favored pseudo 2nd order kinetics.

### 3.3. Adsorption Isotherms

The adsorption isotherms characterize the equilibrium relationship within adsorbate quantity in aqueous and solid phases. Moreover, isotherms determine the adsorbate’s affinity towards adsorbent by evaluating its adsorption capacity [44]. Experiments were conducted at pH 6 and adsorbent dose 1 g L^−1^ by varying initial diclofenac concentrations of 50, 75, 100, 200, 300 and 400 mg L^−1^ at different temperatures (i.e., 25, 35, 45 and 60 °C) for each concentration. Four isotherms models, i.e., Langmuir, Temkin, Dubinin–Radushkevich and Freundlich isotherm models were applied to adsorption data for analyzing the nature of adsorption. The Freundlich isotherm model was applied using the following equation:

(3) $$\ln q_{e}=lnK_{f}+\frac{1}{n}lnC_{e}\;$$

where $C_{e}$ the equilibrium concentration, n denotes the adsorption intensity, while $K_{f}$ represents Freundlich constant. 

Langmuir isotherm model was applied by the Equation (4):

(4) $$\frac{C_{e}}{q_{e}}=\frac{1}{bQ_{max}}+\frac{C_{e}}{Q_{max}}$$

where $C_{e}$ is equilibrium concentration, *b* is Langmuir constant, and $Q_{max}$ maximum adsorption capacity.

Dubinin–Radushkevich is commonly applied to indicate mean free energy distribution on heterogeneous surfaces [44] and is expressed as:

(5) $$\ln q_{e}=\ln q_{m}-\beta \varepsilon^{2}$$

Here, “q_e_” is saturation adsorption capacity (mg/g), activity coefficient “β” helps in finding the mean free energy and Ɛ is the Polanyi potential. The mean free energy “*E*” of adsorption per mole of adsorbate and Polanyi potential are given in Equations (6) and (7).

(6) $$\varepsilon =RTln(1+\frac{1}{C_{e}})$$

(7) $$E=\frac{1}{\sqrt{2\beta }}$$

where 𝑅 is the universal gas constant (8.314 J/mol K) and 𝑇 is temperature in Kelvin.

Temkin isotherm model undertakes indirect adsorbate-adsorbate interactions and accounts for linear decrease in heat of adsorption of all molecules in the layer with the increase in surface coverage [44]. Temkin isotherm expression is given as:

(8) qe=BlnA+BlnCe

where, “*A*” and “*B*” are binding constant (L g^−1^) and heat of sorption (J mol^−1^), respectively. R is the universal gas constant and “T” is absolute temperature. “b” is the Temkin isotherm constant given as:

(9) $$B=\frac{\mathrm{RT}}{b}$$

**Figure 7:**
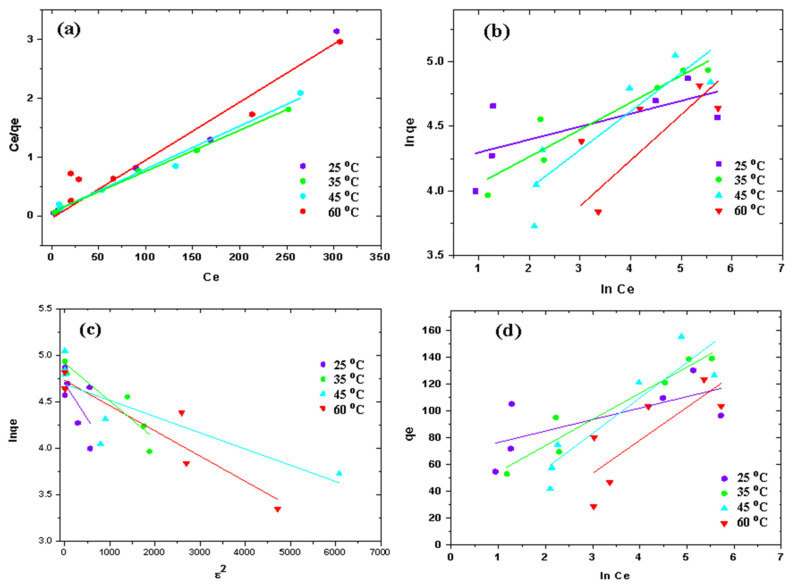
(**a**) Langmuir, (**b**) Freundlich, (**c**) Dubinin–Radushkevich and (**d**) Temkin isotherm for diclofenac adsorption over g-C_3_N_4_/MoO_3_ (3%) nanohybrid.

As shown in Figure 7 and Table 2, the Langmuir isotherm fits better compared to remaining isotherm models. The isotherm results revealed with rise in temperature, the Q_max_ of g-C_3_N_4_/MoO_3_ (3%) nanohybrid also increased, but temperature above 35 °C displayed decrease in the value of Q_max_ which suggested that the adsorption performance is enhanced by increasing the operational temperature up to 35 °C. Temkin model indicated that the heat of sorption “b” (J mol^−1^) decreased with the increase in surface coverage and values of parameter “b” presented in Table 2 infers that increase in temperature enhanced the adsorption capacity thereby increasing surface coverage. The Dubinin–Radushkevich model explains the distribution of the mean free energy of the adsorbate to get adsorbed on the adsorbent’s non-uniform surface. This model presented that the free sorption energy decreased as the solution temperature was externally increased. However, at all studied temperatures the linear plot of Langmuir model gave values of correlation coefficient (R^2^) better than all other applied models. The R^2^ values exhibited by Temkin and Dubinin–Radushkevich model were closer to unity at only 35 and 45 °C, respectively suggesting the optimum temperature range for the adsorption of diclofenac onto the adsorbent. Besides that, adsorption capacities calculated from Langmuir isotherm equation were closer to experimental values and indicating the adsorbent as energetically homogenous where the interaction of diclofenac with g-C_3_N_4_/MoO_3_ (3%) nanohybrid forms monolayer at adsorbent surface. The separation factor RL indicates favorability of adsorption based on the Langmuir equation calculated by:

(10) $$\mathrm{RL}=\frac{1}{1+C_{0}\;b}$$

RL specifies the adsorption type whether it is unfavorable (RL > 1), linear (RL = 1) or favorable (0 < RL < 1). From the above equation, RL value obtained was less than 1 i.e., 0.03 for g-C_3_N_4_/MoO_3_ (3%) nanohybrid. Additionally, RL were found to be favorable for all the isotherms. Figure 6 and Table 2 presented that Langmuir model fits better than other three isotherms in the adsorption data because R^2^ values are near to unity. Isotherm results revealed that with rise in temperature, the Q_max_ of g-C_3_N_4_/MoO_3_ (3%) nanohybrid also increased, but temperature above 35 °C displayed decrease in the value of Q_max_ which suggested that the adsorption performance is enhanced by increasing the operational temperature up to 35 °C.

The Temkin model indicated that the heat of sorption “b” (J mol^−1^) decreased with the increase in surface coverage and values of parameter “b” presented in Table 2 infers that increase in temperature enhanced the adsorption capacity of adsorbent and thereby increasing the surface coverage. The Dubinin–Radushkevich model explains distribution of mean free energy of adsorbate to get adsorb on adsorbent’s non-uniform surface. This model presented that the free sorption energy decreased as the solution temperature was externally increased. However, at all studied temperatures the linear plot of Langmuir model gave values of correlation coefficient (R^2^) better than all other applied models. The R^2^ values exhibited by Temkin and Dubinin-Radushkevich model were closer to unity at only 35 and 45 °C, respectively suggesting the optimum temperature range for the adsorption of diclofenac onto the adsorbent. Besides that, adsorption capacities calculated from Langmuir isotherm equation were closer to experimental values and indicated that the adsorbent was energetically homogenous where the interaction of diclofenac with the g-C_3_N_4_/MoO_3_ (3%) nanohybrid forms the monolayer at the adsorbent surface.

### 3.4. Reusability of g-C_3_N_4_/MoO_3_ (3%) Nanohybrid

The stability and reusability of the spent g-C_3_N_4_/MoO_3_ (3%) nanohybrid was assessed up to the four reuse cycles. After each use the g-C_3_N_4_/MoO_3_ (3%) nanohybrid was dissolved in 15 mL deionized water at pH 4, stirred for 10 min at 600 rpm and filtered, the recovered adsorbent was then calcined at 250 °C to remove residual DCF, washed with de-ionized water and dried in air before next use. Upon repeated use, the nanohybrid maintained its efficiency with minimal loss from 158.2 to 142.6 mg g^−1^ after 4th use, clearly suggesting that the g-C_3_N_4_/MoO_3_ (3%) nanohybrid is very stable and reusable for the removal of diclofenac.

### 3.5. Comparison of g-C_3_N_4_/MoO_3_ (3%) with Other Literature Adsorbents

To estimate the efficiency of the g-C_3_N_4_/MoO_3_ (3%) nanohybrid, its adsorption capacity for diclofenac removal was 3.95-times higher compared with g-C_3_N_4_ at optimized conditions. The highest adsorption capacity of g-C_3_N_4_/MoO_3_ (3%) nanohybrid achieved in this study is compared with previously studies, as shown in Table 3, revealing that g-C_3_N_4_/MoO_3_ (3%) efficiently removes diclofenac from water. However, organobentonite, porous carbon and magnetic amine-functionalized chitosan showed higher adsorption capacities than the g-C_3_N_4_/MoO_3_ (3%) nanohybrid. The difference in activities may be attributed to different adsorbent dosage and operational conditions.

## 4. Conclusions

g-C_3_N_4_/MoO_3_ showed effective removal of diclofenac contaminated water in comparison to base materials (i.e., g-C_3_N_4_ and MoO_3_). While using g-C_3_N_4_/MoO_3_ (3%) nanohybrid 162 mg g^−1^ diclofenac adsorption was achieved at pH 6. The adsorption mechanism could be explained by π–π interaction between aromatic rings of diclofenac and g-C_3_N_4_/MoO_3_ (3%) nanohybrid which is also evident by the FTIR results. Application of different kinetic models revealed that experimental results fit well with pseudo 2nd order kinetics while the Langmuir isotherm explained homogenous adsorption of diclofenac on to g-C_3_N_4_/MoO_3_ (3%) nanohybrid. The linear relation at 45 °C and 35 °C agrees with dubinin-Radushkevich and Temkin isotherms indicated an optimum temperature range of 35–45 °C for the sorption of diclofenac. In this research, the g-C_3_N_4_/MoO_3_ (3%) nanohybrid has appeared as an excellent adsorbent that was well above its environmental concentrations for emerging micropollutants such as diclofenac sodium. This study advocates the application of synthesized adsorbent for the successful elimination of diclofenac micropollutant from aqueous environments to avoid its synergistic toxic effects on human health and environment.

## Figures and Tables

**Table 1:** Kinetics of diclofenac adsorption on to g-C_3_N_4_/MoO_3_ (3%).

Pseudo 1st Order Kinetics				Pseudo 2nd Order Kinetics			
DCF (mg L^−1^)	qe _(exp)_ (mg g^−1^)	qe _(cal)_ (mg g^−1^)	K_1_ (min^−1^)	R^2^	Qe _(cal)_ (mg g^−1^)	K_2_ (min^−1^)	R^2^
Effect of initial pollutant concentration							
50	41.96	1.11	0.0062	0.0784	42.43	0.0241	0.9999
75	66.1	39.4	0.0547	0.9472	67.00	0.0154	1
100	101.61	48.1	0.1029	0.8777	99.11	0.0096	1
200	130	17.51	0.0049	0.5967	130.3	0.0077	0.999
300	158.2	26.6	0.0093	0.4501	155.00	0.0062	0.9977
400	145.10	36.88	0.0053	0.6345	147.00	0.0070	0.9993
Effect of pH Change							
4	44.75	23.68	0.010204	0.5871	44.96	0.0228	0.998
6	158	26.64	0.009379	0.4501	165	0.0066	0.9987
8	132.86	41.5	0.003734	0.8179	132.40	0.0075	0.9987
10	81.12	18.42	0.004386	0.7094	80.94	0.0123	0.9997
Effect of solution temperature							
15	138.81	80.96	0.005558	0.9548	134.88	0.007	0.9972
25	143.71	38.58	0.004168	0.8408	142.5	0.0069	0.9996
35	152.1	75.56	0.008728	0.826	150.4	0.0065	0.9988
45	157.69	68.41	0.010421	0.8403	159	0.0064	0.9994
60	162.58	43.1	0.009336	0.6885	163.89	0.0062	0.9999

**Table 2:** List of calculated parameters from the adsorption isotherm models.

Temperature				
Isotherms	25 °C	35 °C	45 °C	60 °C
**Langmuir** **Isotherm**				
q_m_ (mg g^−1^)	101	142	137	123
b (L mg^−1^)	0.25	0.121	0.124	0.030
R^2^	0.9786	0.9978	0.9838	0.9474
**Freundlich** **Isotherm**				
K_f_	66.54	46.84	30.63	16.8
n_f_	10	4.8	3.4	2.83
R^2^	0.477	0.8879	0.7936	0.5498
**Dubinin-Radushkevich** **Isotherm**				
q_m_ (mg g^−1^)	114	115	137	107
β (mol^2^ kJ^−2^)	0.0001	0.0001	0.0005	0.0016
E (kJ mol^−1^)	70.71	70.71	31.62	17.67
R^2^	0.7239	0.6976	0.931	0.5662
**Temkin** **Isotherm**				
B	8.5666	19.596	26.47	24.681
A (L mg^−1^)	36.124	69.6	35.63	74.77
b (J mol^−1^)	24.26	14.85	14.134	20.2
R^2^	0.4748	0.9303	0.8283	0.6401

**Table 3:** Adsorption capacity of adsorbents for the removal of diclofenac vs g-C_3_N_4_/MoO_3_ (3%).

Adsorbent	Solution pH	Adsorption Capacity (mg g^−1^)	Reference
g-C_3_N_4_/MoO_3_ (3%)	6	162	This study
Goethite	5.23	0.046	[15]
Chitosan/Fe_3_O_4_ composite	6	151	[41]
Functionalized sugarcane bagasse ash	7	0.57	[44]
Organobentonite (OBHDTMA)	7	388	[45]
Porous carbon prepared at 1000 °C (PC-1000)	6.5	392	[46]
Porous Carbon (PC-800)	6.5	186	[46]
CNT/HNO_3_	7	24	[47]
CTAB-ZIF-67	6.5	61	[48]
Activated carbon prepared from olive stones	2	8.8	[49]
Tea waste derived activated carbon	6.47	62	[50]
γ-Fe_2_O_3_ nanoparticles	7	261	[51]
Granular activated carbon	5.5	46.22	[52]
Multi-Walled Carbon Nanotubes	6	19.9	[53]

## Data Availability

Data is contained within the article.

## References

[1] Lonappan L., Brar S.K., Das R.K., Verma M., Surampalli R.Y. (2016). Diclofenac and its transformation products, Environmental occurrence and toxicity-A review. Environ. Int..

[2] McGettigan P., Henry D. (2013). Use of non-steroidal anti-inflammatory drugs that elevate cardiovascular risk, an examination of sales and essential medicines lists in low-, middle-, and high-income countries. PLoS Med..

[3] Acuna V., Ginebreda A., Mor J.R., Petrovic M., Sabater S., Sumpter J., Barcelo D. (2015). Balancing the health benefits and environmental risks of pharmaceuticals, Diclofenac as an example. Environ. Int..

[4] Khan U., Nicell J. (2015). Human health relevance of pharmaceutically active compounds in drinking water. AAPS J..

[5] López-Serna R., Jurado A., Vázquez-Suñé E., Carrera J., Petrovic M., Barcelo D. (2013). Occurrence of 95 pharmaceuticals and transformation products in urban ground waters underlying the metropolis of Barcelona, Spain. Environ. Pollut..

[6] Brozinski J.M., Lahti M., Meierjohann A., Oikari A., Kronberg L. (2012). The anti-inflammatory drugs diclofenac, naproxen and ibuprofen are found in the bile of wild fish caught downstream of a wastewater treatment plant. Environ. Sci. Technol..

[7] Cuthbert R.J., Taggart M.A., Prakash V., Chakraborty S.S., Deori P., Galligan T., Shringarpure R. (2014). Avian scavengers and the threat from veterinary pharmaceuticals. Philos. Trans. R. Soc. B.

[8] Chaudhry M.J.I., Ogada D.L., Malik R.N., Virani M.Z., Giovanni M.D. (2012). First evidence that populations of the critically endangered Long-billed Vulture Gyps indicus in Pakistan have increased following the ban of the toxic veterinary drug diclofenac in south Asia. Bird Conserv. Int..

[9] Prakash V., Bishwakarma M.C., Chaudhary A., Cuthbert R., Dave R., Kulkarni M., Green R.E. (2012). The population decline of Gyps vultures in India and Nepal has slowed since veterinary use of diclofenac was banned. PLoS ONE.

[10] Oaks J.L., Gilbert M., Virani M.Z., Watson R.T., Meteyer C.U., Rideout B., Mahmood S. (2004). Diclofenac residues as the cause of vulture population decline in Pakistan. Nature.

[11] BirdLife International (2017). Gyps indicus. The IUCN Red List of Threatened Species.

[12] Johnson A.C., Dumont E., Williams R.J., Oldenkamp R., Cisowska I., Sumpter J.P. (2013). Do concentrations of ethinylestradiol, estradiol, and diclofenac in European rivers exceed proposed EU environmental quality standards?. Environ. Sci. Technol..

[13] Almoisheer N., Alseroury A., Kumar R., Almeelbi T., Barakat M.A. (2019). Synthesis of graphene oxide/silica/carbon nanotubes composite for removal of dyes from wastewater. Earth Syst. Environ..

[14] Kumar R., Laskar M.A., Hewaidy I.F., Barakat M.A. (2019). Modified adsorbents for removal of heavy metals from aqueous environment, a review. Earth Syst. Environ..

[15] Zhao Y., Liu F., Qin X. (2017). Adsorption of diclofenac onto goethite, Adsorption kinetics and effects of pH. Chemosphere.

[16] Chang E.E., Wan J.C., Kim H., Liang C.H., Dai Y.D., Chiang P.C. (2015). Adsorption of selected pharmaceutical compounds onto activated carbon in dilute aqueous solutions exemplified by acetaminophen, diclofenac, and sulfamethoxazole. Sci. World J..

[17] Qureshia U.A., Hameed B.H., Ahmed M.J. (2020). Adsorption of endocrine disrupting compounds and other emerging contaminants using lignocellulosic biomass-derived porous carbons, A review. J. Water Process. Eng..

[18] Osorio V., Sanchis J., Abad J.L., Ginebreda A., Farre M., Perez S., Barcelo D. (2016). Investigating the formation and toxicity of nitrogen transformation products of diclofenac and sulfamethoxazole in wastewater treatment plants. J. Hazard Mater..

[19] Michael I., Hapeshi E., Osorio V., Perez S., Petrovic M., Zapata A., Fatta-Kassinos D. (2012). Solar photocatalytic treatment of trimethoprim in four environmental matrices at a pilot scale, Transformation products and eco-toxicity evaluation. Sci. Total Environ..

[20] Abdelmelek S.B., Greaves J., Ishida K.P., Cooper W.J., Song W. (2011). Removal of pharmaceutical and personal care products from reverse osmosis retentate using advanced oxidation processes. Environ. Sci. Technol..

[21] Nekouei F., Noorizadeh H., Nekouei S., Asif M., Tyagi I., Agarwal S., Gupta V.K. (2016). Removal of malachite green from aqueous solutions by cuprous iodide–cupric oxide nano-composite loaded on activated carbon as a new sorbent for solid phase extraction, Isotherm, kinetics and thermodynamic studies. J. Mol. Liq..

[22] Xu J., Wu H.T., Wang X., Xue B., Li Y.X., Cao Y. (2013). A new and environmentally benign precursor for the synthesis of mesoporous g C_3_N_4_ with tunable surface area. Phys. Chem. Chem. Phys..

[23] Kumar R., Barakat M.A., Alseroury F.A. (2017). Oxidized g-C_3_N_4_/polyaniline nanofiber composite for the selective removal of hexavalent chromium. Sci. Rep..

[24] Zou Y., Wang X., Ai Y., Liu Y., Ji Y., Wang H., Wang X. (2016). β-Cyclodextrin modified graphitic carbon nitride for the removal of pollutants from aqueous solution, experimental and theoretical calculation study. J. Mater. Chem. A.

[25] Pérez-González M., Morales-Luna M., Santoyo-Salazar J., Crotte-Ledesma H., García-Tinoco P.E., Tomás S.A. (2021). Improved adsorption and photocatalytic removal of Methylene Blue by MoO3 thin films, Role of the sputtering power, film thickness, and sputtering working pressure. Catal. Today.

[26] Rakass S., Hassani H.O., Abboudi M., Kooli F., Mohmoud A., Aljuhani A., Al Wadaani F. (2018). Molybdenum trioxide, efficient nanosorbent for removal of methylene blue dye from aqueous solutions. Molecules.

[27] Wang Y., Xu Y., Wang Y., Qin H., Li X., Zuo Y., Cui L. (2016). Synthesis of Mo-doped graphitic carbon nitride catalysts and their photocatalytic activity in the reduction of CO_2_ with H_2_O. Catal. Commun..

[28] Santos-Beltrán M., Paraguay-Delgado F., García R., Antúnez-Flores W., Ornelas-Gutiérrez C., Santos-Beltrán A. (2017). Fast methylene blue removal by MoO_3_ nanoparticles. J. Mater. Sci..

[29] Cai X., He J., Chen L., Chen K., Li Y., Zhang K., Kong L. (2017). A 2D-g-C_3_N_4_ nanosheets as an eco-friendly adsorbent for various environmental pollutants in water. Chemosphere.

[30] Santos A.L., Reis R.A., Rossa V., Reis M.M., Costa A.L., Veloso C.O., Chiaro S.S. (2012). Silica–alumina impregnated with cerium, nickel, and molybdenum oxides for adsorption of sulfur and nitrogen compounds from diesel. Mater. Lett..

[31] Huang L., Xu H., Zhang R., Cheng X., Xia J., Xu Y., Li H. (2013). Synthesis and characterization of g-C_3_N_4_/MoO_3_ photocatalyst with improved visible-light photoactivity. Appl. Surf. Sci..

[32] Beyki M.H., Mohammadirad M., Shemirani F., Saboury A.A. (2017). Magnetic cellulose ionomer/layered double hydroxide, an efficient anion exchange platform with enhanced diclofenac adsorption property. Carbohyd. Polym..

[33] Li K., Gao S., Wang Q., Xu H., Wang Z., Huang B., Dai Y., Lu J. (2015). In-situ-reduced synthesis of Ti^3+^ self-doped TiO_2_/g-C_3_N_4_ heterojunctions with high photocatalytic performance under LED light irradiation. ACS Appl. Mater. Interfaces.

[34] Li Y., Zhang H., Liu P., Wang D., Li Y., Zhao H. (2013). Crosslinked g-C_3_N_4_/rGO nanocomposites with tunable band structure and enhanced visible light photocatalytic activity. Small.

[35] Wang Y., Zhao S., Zhang Y., Fang J., Zhou Y., Yuan S., Zhang C., Chen W. (2018). One-pot synthesis of K-doped g-C_3_N_4_ nanosheets with enhanced photocatalytic hydrogen production under visible-light irradiation. Appl. Surf. Sci..

[36] Li K., Zeng X., Gao S., Ma L., Wang Q., Xu H., Wang Z., Huang B., Dai Y., Lu J. (2016). Ultrasonic-assisted pyrolyzation fabrication of reduced SnO_2–x_/g-C_3_N_4_ heterojunctions, enhance photoelectrochemical and photocatalytic activity under visible LED light irradiation. Nano Res..

[37] Xie Z., Feng Y., Wang F., Chen D., Zhang Q., Zeng Y., Lv W., Liu G. (2018). Construction of carbon dots modified MoO_3_/g-C_3_N_4_ Z-scheme photocatalyst with enhanced visible-light photocatalytic activity for the degradation of tetracycline. Appl. Catal. B Environ..

[38] Gil A., Taoufik N., García A.M., Korili S.A. (2018). Comparative removal of emerging contaminants from aqueous solution by adsorption on an activated carbon. Environ. Technol..

[39] Zhou X., Dong C., Yang Z., Tian Z., Lu L., Yang W., Chen J. (2018). Enhanced adsorption of pharmaceuticals onto core-brush shaped aromatic rings-functionalized chitosan magnetic composite particles, Effects of structural characteristics of both pharmaceuticals and brushes. J. Clean. Prod..

[40] Head A.R., Tsyshevsky R.V., Trotochaud L., Yu Y., Kyhl L., Lu O.K., Kuklja M.M., Bluhm H. (2016). Adsorption of dimethyl methylphosphonate on MoO_3_, the role of oxygen vacancies. J. Phys. Chem. C.

[41] Pirbazari A.E., Saberikhah E., Badrouh M., Emami M.S. (2014). Alkali treated Foumanat tea waste as an efficient adsorbent for methylene blue adsorption from aqueous solution. Water Resour. Ind..

[42] Hor K.Y., Chee J.M.C., Chong M.N., Jin B., Saint C., Poh P.E., Aryal R. (2016). Evaluation of physicochemical methods in enhancing the adsorption performance of natural zeolite as low-cost adsorbent of methylene blue dye from wastewater. J. Clean. Prod..

[43] Ayawei N., Ebelegi A.N., Wankasi D. (2017). Modelling and interpretation of adsorption isotherms. J. Chem..

[44] Salomao G.R., Pinheiro J.H.P.A., Isique W.D., Torres N.H., Cruz I.A., Ferreira L.F.R. (2019). Diclofenac removal in water supply by adsorption on composite low-cost material. Environ. Technol..

[45] Costa J.S.M., Ramos R.L., Ortega E.P. (2018). Sorption of diclofenac from aqueous solution on an Organobentonite and adsorption of cadmium on organobentonite saturated with diclofenac. Clay Clay Miner..

[46] Mao N., Huang L., Shuai Q. (2019). Facile synthesis of porous carbon for the removal of diclofenac sodium from water. ACS Omega.

[47] Hu X., Cheng Z. (2015). Removal of diclofenac from aqueous solution with multi-walled carbon nanotubes modified by nitric acid. Chin. J. Chem. Eng..

[48] Lin K.Y.A., Yang H., Lee W.D. (2015). Enhanced removal of diclofenac from water using a zeolitic imidazole framework functionalized with cetyltrimethylammonium bromide (CTAB). RSC Adv..

[49] Larous S., Meniai A.H. (2016). Adsorption of Diclofenac from aqueous solution using activated carbon prepared from olive stones. Int. J. Hydrogen Energy.

[50] Malhotra M., Suresh S., Garg A. (2018). Tea waste derived activated carbon for the adsorption of sodium diclofenac from wastewater, adsorbent characteristics, adsorption isotherms, kinetics, and thermodynamics. Environ. Sci. Pollut. R..

[51] Leone V.O., Pereira M.C., Aquino S.F., Oliveira L.C.A., Correa S., Ramalho T.C., Gurgel L.V.A., Silva A.C. (2018). Adsorption of diclofenac on a magnetic adsorbent based on maghemite, experimental and theoretical studies. New J. Chem..

[52] Franco M.A.E., Carvalho C.B., Bonetto M.M., Soares R.P., Feris L.A. (2018). Diclofenac removal from water by adsorption using activated carbon in batch mode and fixed-bed column, isotherms, thermodynamic study and breakthrough curves modelling. J. Clean. Prod..

[53] Hu X., Cheng Z., Sun Z., Zhu H. (2017). Adsorption of diclofenac and triclosan in aqueous solution by purified multi-walled carbon nanotubes. Pol. J. Environ. Stud..

